# Crocin Increases Gastric Cancer Cells’ Sensitivity to Doxorubicin

**DOI:** 10.31557/APJCP.2020.21.7.1959

**Published:** 2020-07

**Authors:** Seyedeh Mahya Shariat Razavi, Reyhaneh Mahmoudzadeh Vaziri, Gholamreza Karimi, Sepideh Arabzadeh, Vahideh Keyvani, Javad Behravan, Fatemeh Kalalinia

**Affiliations:** 1 *Medical Genetics Research Center, Faculty of Medical Sciences, Mashhad University of Medical Sciences, Mashhad, Iran. *; 2 *Department of Cell and Molecular Biology, Kish International Campus, University of Tehran, Kish, Iran. *; 3 *Medical Toxicology Research Center, School of Pharmacy, Mashhad University of Medical Sciences, Mashhad, Iran. *; 4 *Biotechnology Research Center, Pharmaceutical Technology Institute, Mashhad University of Medical Sciences, Mashhad, Iran. *; 5 *Department of Genetics, Faculty of Sciences, Shahid Chamran University of Ahvaz, Ahvaz, Iran. *

**Keywords:** Crocin, multidrug resistance, MDR1, gastric cancer, doxorubicin

## Abstract

**Background:**

Crocin is one of the substantial constituents of saffron extract. It has multiple clinical effects including anti-cancer effects. The development of the multidrug resistance (MDR) phenotype is one of the principal causes of cancer chemotherapy failure. The multidrug resistance protein 1 (MDR1) is one of the MDR-related protein and is often overexpressed in different cancers. In the present study, we aimed to evaluate the influence of crocin on the expression and function of MDR1 protein in EPG85-257 and EPG85-257RDB gastric cancer cell lines.

**Methods:**

The cytotoxicity effect of crocin was evaluated by the MTT assay. The impacts of crocin on the expression and function of MDR1 were assessed by Real-time RT-PCR and MTT assay, respectively.

**Results:**

The results demonstrated that crocin decreased cell viability in a dose-dependent manner with higher intensity on the EPG85-257 than the EPG85-257RDB cells. Crocin did not make any significant changes in the *MDR1 *gene expression level in EPG85-257 and EPG85-257RDB cell lines. In contrast, crocin increased doxorubicin cytotoxicity in drug-resistant cells, which might be induced by reduced MDR1 activity.

**Conclusion:**

In summary, although crocin did not affect mRNA expression of MDR1, results of MTT assay suggest that it might inhibit the MDR1 function.

## Introduction

Nowadays, cancer is the second most important cause of mortality and morbidity worldwide. Gastric cancer is the third leading cause of cancer-related death worldwide (Rugge et al., 2015). Chemotherapy is a conventional treatment for cancer. However, the incidence of multidrug resistance (MDR) to chemotherapy can lead chemotherapy failure in cancer patients (Mitchison, 1998). Gastric cancers show the increasing risk of the occurrence of MDR during the treatment which is the leading cause of gastric cancer chemotherapy failure (Zhang and Fan, 2007).

In MDR phenomena, cancer cells show resistance to different drugs with different functions and structures that are frequently used in chemotherapy. Various mechanisms involved in MDR phenomena such as decrease in drug harvesting, decrease in drug activity, and increase in drug efflux of the cell. ATP-binding cassette (ABC) transporters are the main factors that play a role in MDR phenomena through drug efflux. They are transmembrane proteins that pump several substances, including cytotoxic reagents, out of the cell membrane (Samuel et al., 2017). Multidrug resistance protein1 (MDR1) belongs to the ABCB transporters subfamily and is involved in drug resistance of various types of cancer cells (Holohan et al., 2013). MDR1 is usually expressed in epithelial cells and is active intensively in the blood-brain barrier. MDR1 has various substrates that make it a prevalence transporter in MDR (Löscher and Potschka, 2005). 

Over the past decades, various studies introduced novel herbal substances as anti-cancer candidates, that leads to developing more effective and safer cancer treatments (Safarzadeh et al., 2014). One of the main components in the *Crocus sativus L*., which is commonly known as saffron, is a glycosylated carotenoid named crocin (C22H44016). Different studies indicated various medicinal effects of crocin, such as protecting myocardial cells against hypoxia (Wu et al., 2010), anti-atherosclerosis (He et al., 2005), anti-inflammatory (Nam et al., 2010), antioxidant (Asdaq and Inamdar, 2010), anti-anxiety (Pitsikas et al., 2008), anti-depressant (Lopresti and Drummond, 2014) and enhancement of sexual instinct (Hosseinzadeh et al., 2008). Crocin has neuroprotective effects against traumatic brain injury through activation of notch signaling pathway, which can act as oncogene or tumor suppressor in different cell types (Wang et al., 2015; Razavi et al., 2019). Moreover, there are many studies which showed the anti-cancer effects of crocin against breast, leukemia, bladder, cervical and colorectal cancer cells (Zhao et al., 2008a; Xu et al., 2010; Amin et al., 2015; Hire et al., 2017; Mollaei et al., 2017).

Different studies suggest the possibility of a relationship between crocin and regulation of MDR transporters. Crocin inhibited brain microglial cells liberation of nitric oxide (Noureini and Wink) which had been induced by Lipopolysaccharides (LPS) and reduced the production of interleukin-1beta (IL-1β), tumor necrosis factor-alpha (TNFα) and reactive oxygen species (Abolhoda et al.) that lead to the decrease of NF-kappa B activation (Mosaffa et al., 2009). Furthermore, it has been shown that the anti-inflammatory drugs induced the production of ROS (Abolhoda et al.) and caused oxidative stress to arise that finally lead to an increase in the MDR association protein expression in colorectal cancer cells (Tatebe et al., 2002). Crocin was shown to down-regulate the expression of multidrug resistance-associated protein (MRP) and decrease drug resistance in a cisplatin-resistant ovarian cell line (Mahdizadeh et al., 2016b). In the current study, we aimed to evaluate the effect of crocin administration on the expression and function of MDR1 in EPG85-257 gastric cancer cell line and its drug-resistant derivative EPG85-257RDB cells.

## Materials and Methods

*Material*

RPMI 1640 with L-glutamine and fetal bovine serum (FBS) were bought from Gibco (USA). Penicillin/streptomycin, MTT, DMSO (Dimethyl Sulfoxide), and doxorubicin were acquired from Sigma-Aldrich, Germany. Crocin was kindly received from Dr. Seyed Ahmad Mohajeri (Pharmaceutical Research Center, Mashhad University of Medical Sciences, Iran). RNA TriPure Isolation kit was purchased from Roche Applied Science, Germany. One-Step SYBR PrimeScriptTM RT-PCR Kit II was obtained from Invitrogen, USA. EPG85-257 and EPG85-257RDB gastric cancer cell lines were generously received from Professor H. Lage (Molecular Pathology Department, Charite Campus Mitte, Berlin, Germany).

*Crocin solution preparation*

Crocin was extracted from saffron using crystallization method; its purity was tested by HPLC that was more than 96% pure (Hadizadeh et al., 2010). As the molecular weight of crocin is 976.972 g/mol, 0.001 gr of crocin was dissolved in 10 μl of DMSO (dimethyl sulfoxide) (Cinnagen, Iran) and brought to 1 ml final volume by adding phosphate-buffered saline (PBS). The final concentration of dissolved crocin was 1,024 mM and it was stored at −20°C. Different working concentration of crocin (10, 20, 40, 60, 80, and 100 μM) was freshly prepared before starting each experiment by adding cell culture medium to it. In order to prepare the doxorubicin stock solution (5 µM), 0.43 μl of 3440 µM doxorubicin (Pars Darou, Iran) was brought to 300 μl by adding complete culture medium. 

*Cell culture and treatment*

The cells were cultured in RPMI-1640 supplemented by 10% (v/v) FBS and 1% penicillin/streptomycin and were kept at 37°C in a humidified air containing 5% CO_2_. Gastric cancer cell lines were treated with crocin (0–100 μM), doxorubicin (0–500 nM) and different concentrations of crocin (0–100 μM) and doxorubicin (0–500 nM) simultaneously for 24-72 h.

*MTT cytotoxicity assay*

Drug sensitivity of the EPG85-257 and EPG85-257RDB cell lines were assessed by MTT (3-(4, 5-dimethylthiazol-2-yl)-2, 5-diphenyl tetrazolium bromide) assay. Cells were seeded at an initial density of 104 cells/well in 96-well plates and incubated at 37 °C in a 5% CO_2_ atmosphere. After 24 h, the culture medium was exchanged with different concentrations of crocin (0–100 μM), and doxorubicin (0-500 nM) in a final volume of 100 μl of growth medium and the cells were incubated for 24, 48 and 72 h. DMSO was used at the same volume of a test compound as solvent control. On the final day, in order to avoid the interference of crocin color with the MTT color, the culture medium was removed, and the cells were carefully washed with PBS to ensure that crocin was completely removed. Then, the cell viability was analyzed by adding the culture medium containing 0.5 mg/ml MTT to the cells. After four hours of incubation, the reduced MTT dye (formazan crystals) was solubilized by adding 100 μl of DMSO. Subsequently, the absorbance of formazan produced by living cells was analyzed by ELISA plate reader (BioTek, Bad Friedrichshall, Germany) with a test and reference wavelength of 550 and 630 nm, respectively. The ratio of OD test/OD control was used to determine the percentage of the cell proliferation. Each test was accomplished in triplicate and repeated at least three times.

After identifying the half-maximal inhibitory concentration (IC50) of crocin and doxorubicin, EPG85-257 and EPG85-257RDB cells were treated simultaneously with various concentrations of crocin (0–100 μM) and doxorubicin (0–500 nM) and incubated at 37°C in a 5% CO_2_ atmosphere for three days to study the effect of crocin on doxorubicin cytotoxicity.

*Real-Time RT-PCR*

In order to evaluation the effects of crocin on the expression of MDR1, the cells were treated with different concentrations of crocin for 48 h. Then, the total RNA was extracted by RNA TriPure Isolation kit and its concentration was specified by the NanoDrop 1000 spectrophotometer (Thermo Fisher, Scientific, Wilmington, DE). Comparative quantitative Real-time reverse transcription-PCR was done using One-Step SYBR PrimeScriptTM RT-PCR Kit II and real-time cycler Mx3000P™ Stratagen (Stratagen, USA) to evaluate *MDR1* expression level in gastric cancer cell lines. Beta-actin was used as a normalizer gene. The sequences of primers were as following: MDR1 Forward: 5’- CCCATCATTGCAATAGCAGG-3’and MDR1 Reverse: 5’- TGTTCAAACTTCTGCTCCTGA-3’ (Taheri et al., 2017); Beta-actin Forward: 5’-TCATGAAGTGTGACGTGGACATC-3’ and Beta-actin reverse 5’-CAGGAGGAGCAATGATCTTGATCT-3’(Kalalinia et al., 2012; Kalalinia et al., 2014). cDNA synthesis was performed at the beginning of the process (50°C for 5 min), and RT-PCR reaction was performed according to manufacturer’s protocol: 95°C for 2 min and PCR amplification cycles (40 cycles at 95°C for 15 s, 60°C for 1 min). The relative expression of *MDR1 *gene were reported as the ratio of target/reference gene expression in the EPG85-257RDB cells divided by the ratio of target/reference gene expression in the *EPG85-257* cells. 

*Statistical analysis*

Results were reported as mean ± SD. All experiments were performed at least three times independently. Statistical analysis was done by SPSS version 16.0 using ANOVA, with Tukey’s posthoc test. P values under 0.05 considered as significant.

## Results

*Effects of crocin on the proliferation rate of gastric cancer cell lines*

**Figure 1:**
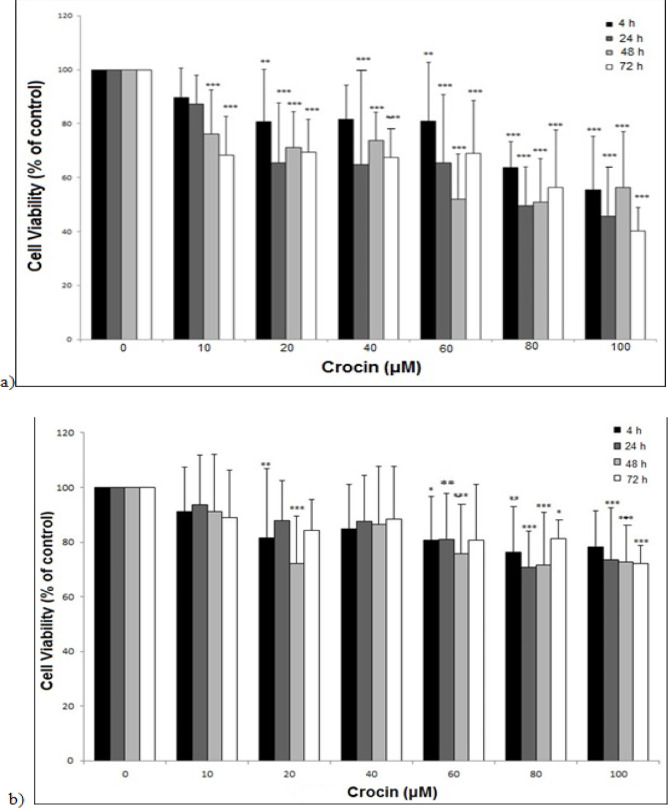
The Effects of Crocin on the Cell Viability of EPG85-257 (a) and EPG85-257RDB (b) cell lines. The cells were incubated with various concentrations of crocin at 37 °C for 4, 24, 48 and 72 h. Cell viability was measured by the MTT assay. Each experiment was repeated independently three times in triplicate tests and data are shown as mean ± SD. *P ≤ 0.05; **P ≤ 0.01; ***P ≤ 0.001

MTT cytotoxicity assay was performed to discover the effects of crocin on the cell survival of EPG85-257 and EPG85-257RDB cell lines. The results displayed that crocin significantly reduced the survival of the EPG85-257 cell line in a concentration-dependent manner (Figure 1a). The half-maximal inhibitory concentrations (IC_50_) of crocin after treatment for 24, 48, and 72 h, were 83, 78, and 80 μM, respectively. A similar inhibitory pattern with less intensity was observed in the EPG85-257RDB cells (Figure 1b). None of the crocin treatments in the drug-resistant cell line could decrease cell viability by more than 50 percent.

*MDR1 mRNA expression in the EPG85-257 and EPG85-257RDB cells*

**Figure 2:**
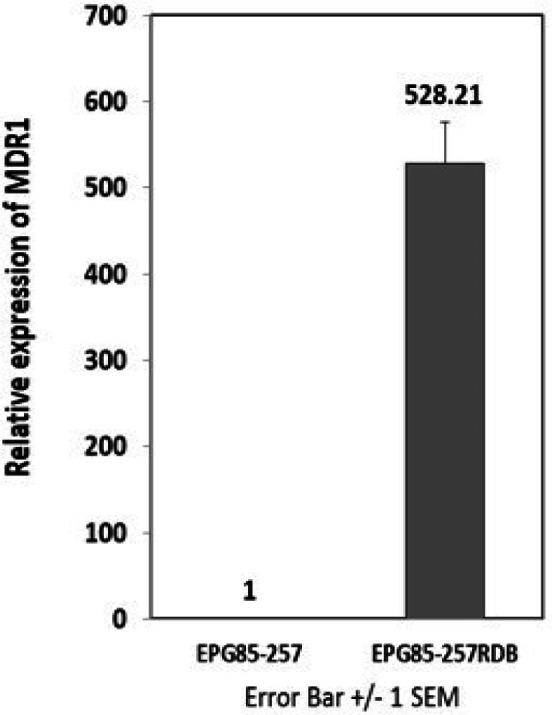
Basal Expression of MDR1 in Gastric Cancer Cell Lines was Analyzed by Real Time RT- PCR. MDR1 mRNA level of EPG85-257RDB drug resistance cell line was compared to EPG85-257 parental drug sensitive cells. Real-time RT-PCR was performed on total RNA and normalized to β-actin. Values is shown as mean ± SD (n = 3).

Real-time RT-PCR was used to measure the differences in the MDR1 mRNA expression level in EPG85-257RDB drug-resistant cells compared to EPG85-257 parental drug-sensitive cells. As shown in Figure 2, the MDR1 mRNA expression level in EPG85-257RDB cells was 528 times more than EPG85-257 cells.

*The effects of crocin on MDR1 mRNA expression in EPG85-257 cell lines*

**Figure 3:**
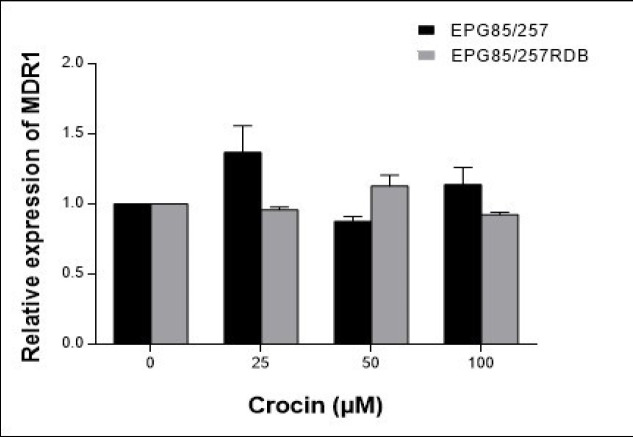
The Effects of Crocin on the MDR1 mRNA Expression Levels in the EPG85-257 and EPG85-257RDB Cell Lines. Cells were treated for 48 h with crocin (0–100 μM), and MDR1 gene expression was measured by real-time RT-PCR using total RNA extracted from control and treated cells. Values were normalized to the β-actin content of each samples. The results were expressed as the ratio of target/reference of the treated samples divided by the ratio of target/reference of the untreated control sample and expressed as mean ± SEM (n = 4).

The cells were treated with several concentrations of crocin (0–100 μM) for 48 h to assess the expression level of *MDR1 mRNA* using Real-time RT-PCR method. The results showed that the expression of the *MDR1* gene did not change significantly within 48 h treatment with crocin in parental and resistance tested cell lines (Figure 3). 

*The effects of crocin on the MDR1 transporter activity*

**Figure 4:**
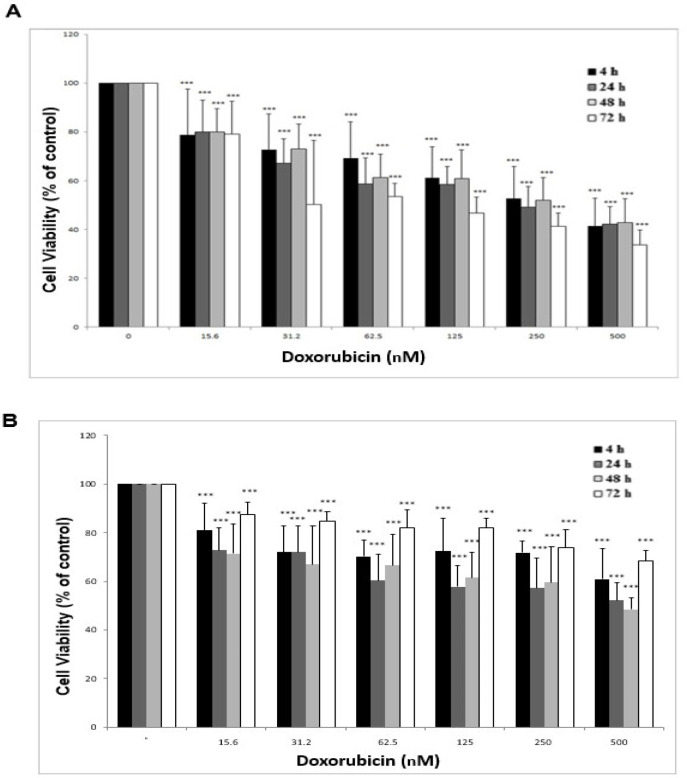
Viability Assay of EPG85-257 (a) and EPG85-257RDB (b) cell lines under the treatment with doxorubicin. The cells were incubated with different concentrations of doxorubicin (0–500 nM) for 4, 24, 48 and 72 h. Cell viability was measured by the MTT assay. Each experiment was repeated independently three times in triplicate tests and data are shown as mean ± SD. ***P ≤ 0.001

The sensitivity of gastric cancer cell lines to the doxorubicin (substrate of MDR1 pump) was analyzed in the presence or absence of different concentrations of crocin to explore the effects of crocin on the MDR1 transporter activity. At first, the IC_50_ value of doxorubicin on EPG85-257 and EPG85-257RDB cells were determined by treating the cells with several concentrations of doxorubicin (0–500 nM) for 24, 48, and 72 h. It was observed that the survival rate of the EPG85-257 cells that were treated with several concentrations of doxorubicin had a significant decrease compared to the control cells (doxorubicin with zero concentration) in a time and concentration-dependent manner (Figure 4a). Also, the viability of EPG85-257RDB cell line was decreased significantly after 24 and 48 h treatment with doxorubicin in a concentration-dependent manner while this decreased was observed with less intensity after 72 h (Figure 4b). These anti-cell viability effects of doxorubicin were observed with a higher intensity in the parental EPG85-257 cells compared to resistance EPG85-257RDB cells. The IC_50_ values of doxorubicin in EPG85-257 and EPG85-257RDB cell lines were shown in Table 1.

**Figure 5:**
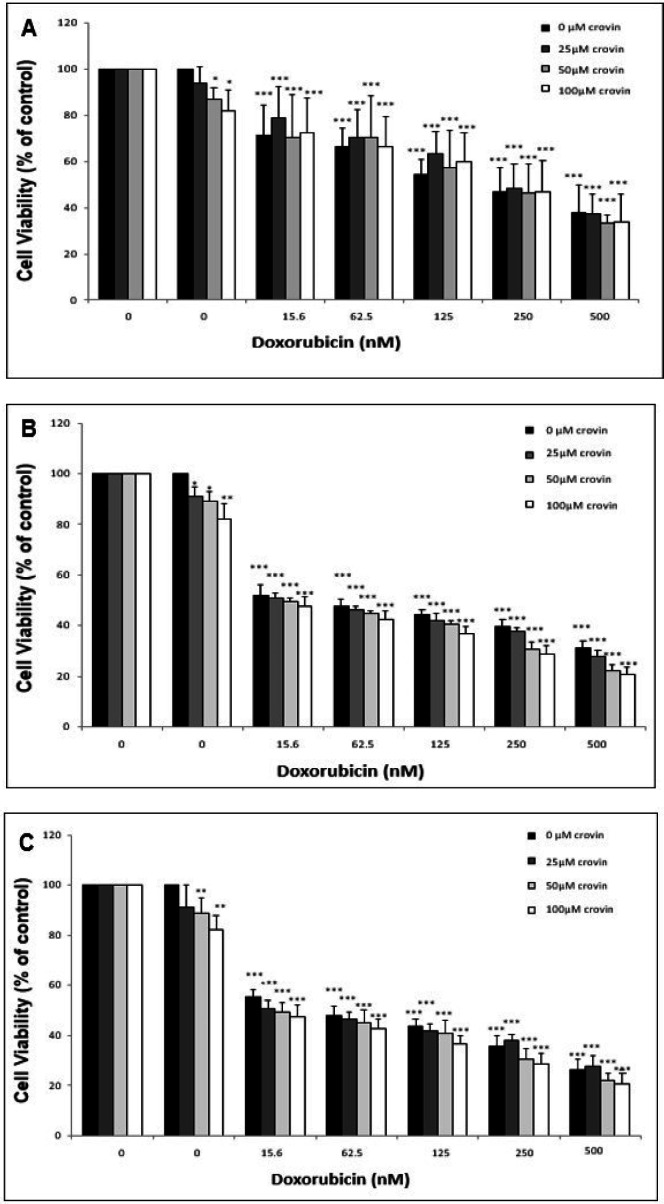
The Cytotoxicity Effects of Cotreatment of the EPG85-257 Cells with Crocin and Doxorubicin was Analyzed by MTT Assay. EPG85-257 cells treated with different concentrations of crocin and doxorubicin for 24 hours (A), 48 hours (B) and 72 hours (C). The results are expressed as mean ± SD (n = 3); *, *p* < 0.05; **, *p*< 0.01; ***, *p* < 0.001

**Figure 6.**
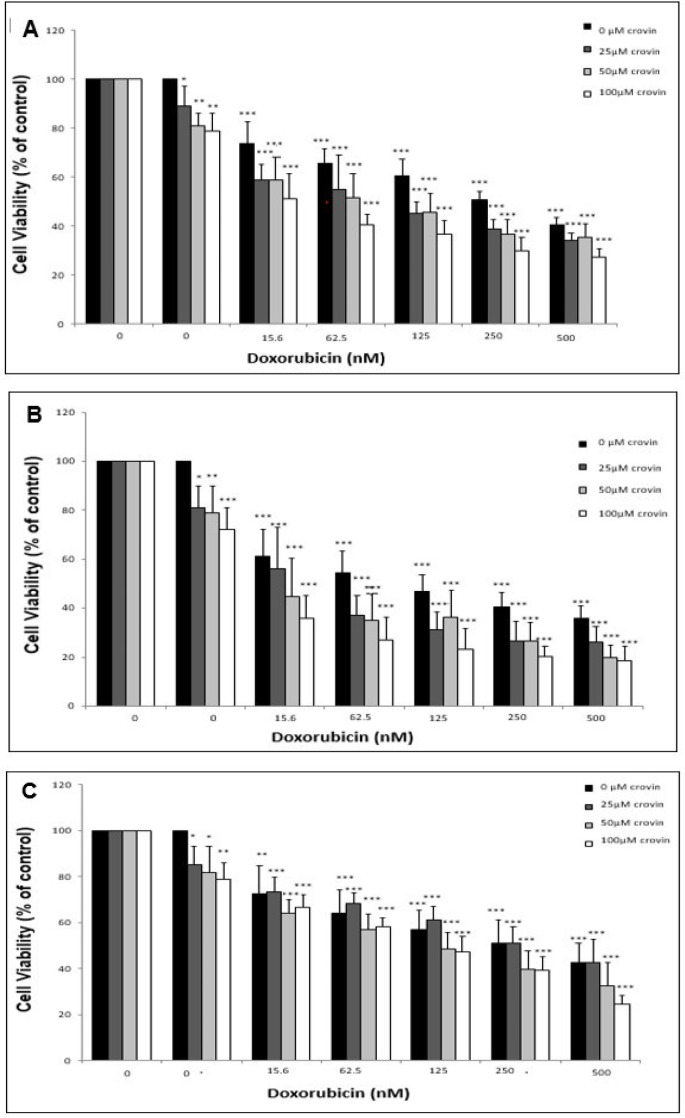
The Cytotoxicity Effects of Cotreatment of the EPG85-257 RDB Cells with Crocin and Doxorubicin was Analyzed Using MTT Assay. EPG85-257 cells treated with different concentrations of crocin and doxorubicin for 24 hours (A), 48 hours (B) and 72 hours (C). The results are expressed as mean ± SD (n = 3); *, *p* < 0.05; **, *p*< 0.01; ***, *p* < 0.001

In the next step, the cells were treated simultaneously with different concentrations of crocin (0, 25, 50 and 100 μM) and doxorubicin (0, 15.6, 62.5, 125, 250 and 500 nM) to measure the impact of crocin on the cytotoxicity of doxorubicin. The viability of the cells was evaluated in different incubation times (24, 48, and 72 h) using MTT assay. It was observed that the crocin treatment lead to a slight decrease in cell viability of EPG85-257 cells that has been simultaneously treated with doxorubicin (Figure 5). Furthermore, the results of MTT assay showed that different concentrations of crocin increased cytotoxicity of doxorubicin in the EPG85-257RDB cell line at different times (Figure 6). The reduction of cell viability was more intensive and mainly dependent on crocin concentration at 24 and 48 hours. 

**Table 1:** IC_50_ Value of Doxorubicin in EPG85-257 Cell Lines in Different Duration Time

Duration of doxorubicin treatment (hours)	IC_50_ (nM) EPG85-257 cell line	IC_50_ (nM) EPG85-257RDB cell line
24	320	435
48	341	415
72	266	---------

## Discussion

Multidrug resistance is a key reason for chemotherapy inefficiency in cancer treatment. One of the main molecular mechanisms involved in multidrug resistance is an enhanced efflux of the drugs by ABC transporters, such as MDR1 (multidrug resistance protein1), that are expressed on the surface of the cells and pump chemotherapy drugs out of the cell. Many studies tried to find the specific inhibitors of ABC transporters to fade drug resistance phenomena in cancers (Li et al., 2016). Crocin has various anti-cancer activities in different cancer cells (Zhao et al., 2008; Xu et al., 2010; Bolhassani et al., 2014), and could be used for cancer treatment. In our study, we evaluated the effects of crocin on the expression level and function of MDR1 in the human gastric cancer cell line EPG85-257 and its drug-resistant derivative cell line (EPG85-257RDB). 

Doxorubicin is a substrate for MDR1 transporter (Abolhoda et al., 1999), and a positive correlation between MDR1 expression and doxorubicin resistance in human cancer cell lines has been shown, previously (Mechetner et al., 1998). Our studies demonstrated that *MDR1* gene expression in EPG85-257RDB, which is a multidrug-resistant human gastric carcinoma cell line, is about 528 times more than its parental drug-sensitive EPG85-257 cell line. It was also shown that the cytotoxicity effect of doxorubicin was more intensive in EPG85-257 cells in compared with EPG85-257RDB cells. According to these results, it seems that the higher expression of MDR1 in EPG85-257RDB cells leads to higher efflux of doxorubicin and less cell cytotoxicity effect of it. Furthermore, similar results were observed when the cells were treated with crocin. The inhibitory effect of crocin on the cell viabilty was more intensive in the parental EPG85-257 cells than EPG85-257RDB cells. It may be concluded that crocin might act as a substrate for MDR1 transporter. 

Various studies focused on the molecular mechanism of anti-cancer activity of crocin in different human cancer cells. In a study on bladder cancer T24 cell line, cDNA microarray analyses showed that expression of 836 genes was changed after crocin treatment. Most of the genes encoded the proteins that are essential for the regulation of cell cycle and cell apoptosis, or they act as transcription factors (Lv et al., 2008). Crocin could arrest the cell cycle and leads to cell apoptosis through inhibiting the expression of cyclin D1, Bcl-2, survivin, and LDHA (lactate dehydrogenase A) (Zhao et al., 2008; D’Alessandro et al., 2013). It can also affect tumor cell cycle and apoptosis by increasing the activity and expression of Nrf2 (nuclear factor erythroid 2-related factor 2) and Bax (Hoshyar et al., 2013; Kim et al., 2014). The results of a study on HepG2 cells showed that crocin had an inhibitory effect on telomerase activity, which can be a reason for its anti-proliferative role (Noureini and Wink, 2012). Crocin, in combination with cisplatin, could suppress invasion and induce apoptosis (Li et al., 2013). Proteomic analysis revealed that crocin binds to different cell membrane proteins like membrane transporters and enzymes that have a role in signal transduction and ATP homeostasis (Hosseinzadeh et al., 2014).

Moreover, the inhibitory effect of crocin as a carotenoid, among other carotenoids, such as β-carotene, retinoic acid, canthaxanthin, on MDR1 (P-glycoprotein) had been investigated. Crocin decreased MDR1 expression in the T lymphoblast CCRF-CEM cell line and could act as an adjuvant to sensitize cells to chemotherapy (Eid et al., 2012). Similarly, in a study on the ovarian cancer cell line, the effect of crocin on the expression and function of MRP1 and MRP2 (Multidrug resistance-associated protein 1), other sub-family of ABC transporters, was examined. Crocin significantly reduced the expression level of MRP1 and MRP2 (up to about 50% and 60% respectively) in A2780/RCIS cells. It increased doxorubicin cytotoxicity in A2780/RCIS cells significantly but did not have an impact on A2780 cells (Mahdizadeh et al., 2016). Compared to previous studies, it can be concluded that the crocin impact and its severity on the receptor expression and activity may depend on the type of cancer cell and receptor.

In the present study, we investigated the effects of crocin on MDR1 expression and activity. In order to achieve this goal, the sensitivity of gastric cancer cell lines to doxorubicin in the presence or absence of nontoxic concentrations of crocin were analyzed. The MDR1 activity assay was performed in two different ways. First, the short-time effect was analyzed by MTT assay after the treatment of the cells with crocin to determine the direct interaction of crocin and transporters. Second, the long-time effect was assayed by real-time RT- PCR to evaluate crocin indirect effects on transporters’ gene expression. Analysis of the short-time effect of crocin showed that it significantly increased the sensitivity of EPG85-257RDB cells to doxorubicin in a concentration-dependent manner. On the other hand, it was observed that crocin has no long term effect on MDR1 by changing its expression. Our results indicate that crocin can increase cell sensitivity to doxorubicin, and it can be used as adjuvants in cancer chemotherapy. 

In the current study, we aimed to investigate the ability of crocin on inhibiting multidrug resistance in human gastric cancer cell lines by restricting the function of MDR1 transporter. Our results showed that crocin could not change the *MDR1* gene expression. It seems that crocin can increase the cytotoxicity of doxorubicin in the human EPG85-257 and EPG85-257RDB gastric cell lines. Further studies are recommended to clarify if using chemotherapeutics in combination with crocin could be an effective method to increase chemotherapy efficacy and reduce the effect of MDR in gastric cancer treatment.
